# Encoding microcarriers for biomedicine

**DOI:** 10.1002/SMMD.20220009

**Published:** 2023-02-14

**Authors:** Xiaowei Wei, Yixuan Shang, Yefei Zhu, Zhuxiao Gu, Dagan Zhang

**Affiliations:** ^1^ Laboratory Medicine Center The Second Affiliated Hospital of Nanjing Medical University Nanjing China; ^2^ Department of Clinical Laboratory Institute of Translational Medicine The Affiliated Drum Tower Hospital of Nanjing University Medical School Nanjing China

**Keywords:** bionic organ chip, biosensing, cell capture, cell culture, drug delivery, drug screening, encoding microcarrier, multiplexed analysis

## Abstract

High throughput biological analysis has become an important topic in modern biomedical research and clinical diagnosis. The flow encoding scheme based on the encoding microcarriers provides a feasible strategy for the multiplexed biological analysis. Different encoding characteristics invest the microcarriers with different encoding mechanisms. Biosensor analysis, drug screening, cell culture, and the construction and evaluation of bionic organ chips can be realized by decoding the microcarriers and quantifying the detection signal intensity. In this review, the encoding strategy of microcarriers was divided into the optical and non‐optical encoding approaches according to their encoding elements, and the research progress of the microcarrier encoding strategy was elaborated. Finally, we summarized the biomedical applications and predicted their future prospects.

## Graphical abstract

According to the encoding principle, microcarrier encoding strategies are divided into optical encoding methods and non‐optical encoding methods. The research progress of microcarrier encoding in biomedicine was reviewed. Based on this, we constructed and evaluated the applications of encoded microcarriers with different mechanisms in biosensing analysis, drug screening, cell culture and bionic organ microarray, and prospected their future development prospects.

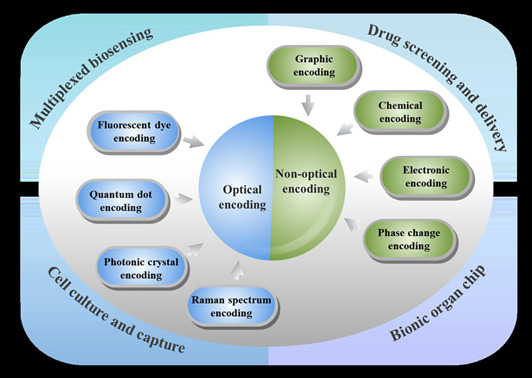

1

**Key points**

- A systematic review of microcarrier‐based encoding strategies.
- Different encoding principles: optical encoding and non‐optical encoding were described and discussed elaborately.
- Comparison of traditional planar microarray and microcarrier high‐throughput encoding in biological analysis.
- Review of the biomedical applications based on encoding microcarriers, including multiplexed biosensing, drug screening and delivery, 3D cell culture, and bionic organ chips.

## 2 INTRODUCTION

With the development of clinical diagnosis and treatment, molecular‐level bioanalytical techniques have attracted extensive attention.^1^, ^2^, ^3^, ^4^ Particularly, as an important research trend, disease‐related biomarker analysis has achieved extensive development in modern clinical diagnosis. However, in practical detection, the single biomarker results are often insufficient to provide specific and accurate evidence for clinical diagnosis. Simultaneous detection of multiple biomarkers brings new challenges to laboratory diagnostic techniques. Under such a demand, a multiplexed detection technique was proposed to replace the traditional detection technique.^5^, ^6^ In recent years, this multiplexed detection technique has developed rapidly in laboratory diagnosis. On the premise of ensuring high detection sensitivity, it can simultaneously perform multi‐channel parallel analysis of diverse disease‐related biomarkers, while possessing outstanding advantages such as simple operation, rapidity, and high throughput.^7^, ^8^ In addition, with the rapid development of the human genome project and molecular biology in the last century, microarray technology has been proposed as a typical solid‐phase chip‐based multiplex analysis strategy. Based on this technology, multiple differential analysis units are constructed on the surface of a solid chip, and then diverse probe molecules are fixed in different locations and arranged in an ordered lattice. Therefore, after responding to the target molecular, the qualitative and quantitative analysis of the disease‐related biomarkers can be realized by reading the array of information of the positive site, including the position and signal strength. This multiplexed analysis scheme met the demand of the medical laboratory for high‐throughput detection, which greatly promoted the development of clinical diagnostics.^9^, ^10^, ^11^, ^12^, ^13^, ^14^, ^15^, ^16^ However, the bioanalysis efficiency of the solid‐liquid hybridization of target molecules and microarray bases was not ideal. In addition, functional solid substrates tend to adsorb some charged biological molecules, which affects the repeatability of multiplexed detection.^17^, ^18^, ^19^, ^20^ Therefore, the development of novel high‐throughput analysis strategies is highly anticipated.

Multiplexed analysis based on the encoding microcarriers is a feasible emerging high‐throughput analysis strategy.^21^, ^22^, ^23^, ^24^, ^25^ Microcarriers have a unique encoding performance, which can be used to identify and distinguish the different microcarriers in biological analysis. A specific probe molecule is fixed on the surface of each encoding microcarrier. When the target molecule is present in the detection system, the encoding microcarrier fixed with the specific probe can capture the corresponding target biomarker and display the detection signal. The encoding microcarrier with specific signal intensity could realize the analysis of various biomarkers, as the laboratory evidence for disease diagnosis. Compared with the position encoding scheme of the solid phase microarray, the microcarriers based on the floating encoding principle can move freely in the solution and capture free biomarkers in the sample. This analysis strategy can significantly shorten the reaction time and thus improve the bioanalysis efficiency. In addition, the encoding microcarriers are mostly micron or nanoscale particles. Benefiting from the smaller size and the larger specific surface area, they can couple more probe molecules, effectively increasing the detection sensitivity. These advantages make the encoding microcarriers suitable for the quantification of trace biomarkers, and only require tiny amounts of clinical samples.^26^, ^27^, ^28^, ^29^, ^30^ In conclusion, encoding microcarriers show great application potential in the biomedical field.^31^, ^32^

**FIGURE 1:**
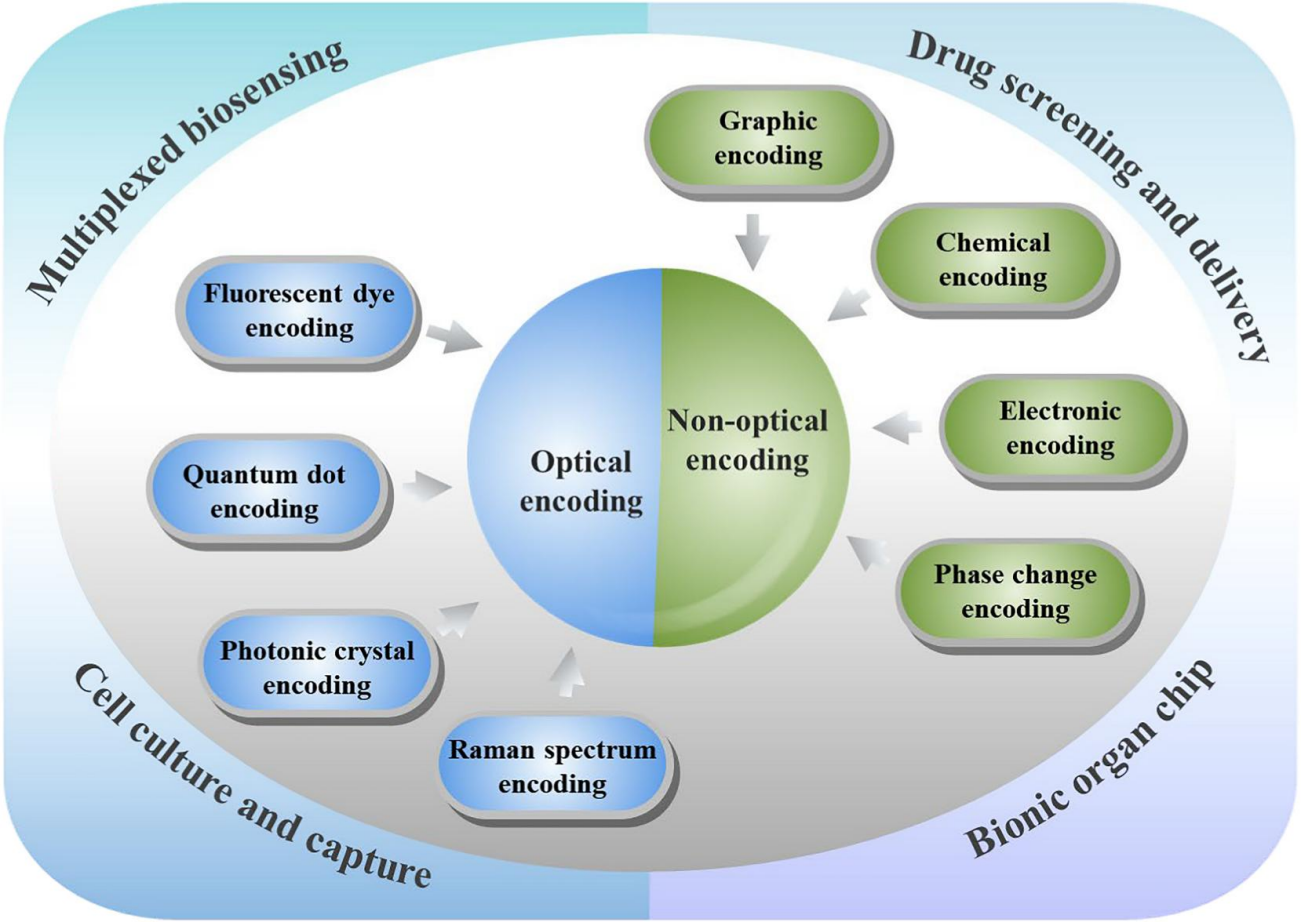
Schematic illustration of encoding microcarriers for biomedical applications

Although the encoding microcarrier technology has made the breakthrough progress at present and shown a broad application prospects in many fields,^33^ few literature have systematically reviewed its biomedical application. Therefore, according to the classification of microcarrier encoding elements (Figure 1), we give a review and prospected research trend on the application progress of various encoding strategies in the biomedical field.

## 3 ENCODING STRATEGIES OF MICROCARRIERS

The encoding strategies of microcarriers are mainly divided into optical encoding and non‐optical encoding. Fluorescent dyes, quantum dots, photonic crystals, and Raman spectra are all encoding elements based on optical mechanisms, which have been widely used due to the significant advantage of large encoding numbers. Meanwhile, the encoding pathways of graphics, chemistry, electronics, and phase transitions are based on non‐optical mechanisms. These multiplexed encoding strategies have important research and application significance in the biomedical field.

### 3.1 Optical encoding

#### 3.1.1 Fluorescent dye encoding

**FIGURE 2:**
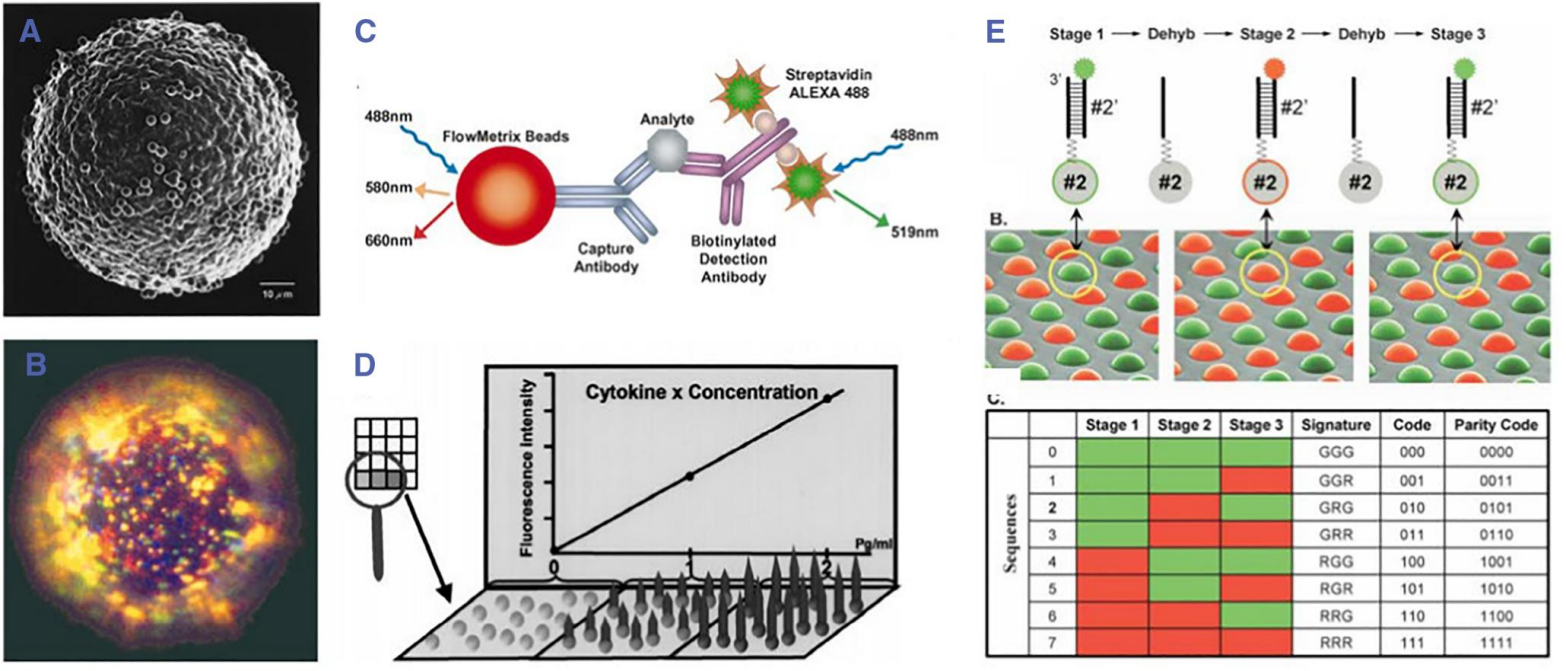
(A, B) Scanning electron microscopy (Reproduced with permission.^35^ Copyright 2000, American Chemical Society) and fluorescence characterization (Reproduced with permission.^36^ Copyright 2001, John Wiley and Sons) of fluorescent dye encoded microspheres. (C) Schematic diagram of multiplexed analysis of cytokines based on Flow Metrix E technology. Reproduced with permission.^37^ Copyright 2000, Elsevier. (D) Schematic diagram of a cytokine planar microarray based on Luminex‐100. Reproduced with permission.^37^ Copyright 2000, Elsevier. (E) Schematic diagram of multiplexed encoding of bicolor labeled nucleic acid. Reproduced with permission.^39^ Copyright 2004, Cold Spring Harbor Laboratory Press.

The fluorescent dye encoding strategy is to modify the fluorescent dye on the surfaces of the encoding microcarriers using physical or chemical methods and then identify the fluorescence signal with a fluorescence microscope to analyze the type and number of the coupled probe molecules. Trau et al. fixed the fluorescent dye label particles on the surface of the microcarrier through physical adsorption and analyzed the encoded markers on the microcarrier through a fluorescence signal to decode the biomolecular reactions (Figure 2A).^34^, ^35^ Trau’s group improved the scheme in the later stage by making the silicon dioxide microcarrier coated with multi‐layer fluorescent molecular dye using the covalent bonding method to prepare the encoding microcarriers (Figure 2B).^36^ Compared with the encoding strategy based on physical adsorption, the improved scheme was more stable. Luminex (Figure 2C,D)^37^ prepared the encoding microspheres by using a fixed proportion of fluorescent molecular dyes, and the encoding amount depended on the dye color type and proportion used. Although the encoding microcarriers were batch prepared, the dye molecules used for fluorescence encoding were unstable, which makes the fluorescence signal easily quenched under high temperature or strong ultraviolet radiation. In addition, the overlapping interference between excitation and emission spectra of various fluorescent molecular dyes limited the selection of fluorescent dyes and the practical application of fluorescent encoding.

Some researchers modified the capture probes on the surface of microcarriers to identify the target molecules in biological reactions. Once the target molecule was captured, it could be decoded and detected by the fluorescent‐labeled probe. Luo et al. prepared a DNA molecular probe labeled with a fluorophore and realized multiplexed encoding by regulating the color and number of fluorophores.^38^ Illumina’s BeadArray technology modified the fluorescent molecular dyes on the nucleic acid encoding sequences and coupled them on the surface of microcarriers (Figure 2E).^39^ Fluorescent dye was used for microcarrier encoding, while probe sequence was used for the hybridization reaction to detect nucleic acid molecules. This analysis system has excellent encoding characteristics and high throughput, which can be used for gene expression profiling.

Although the fluorescent encoding technology has been widely used, the application of fluorescent encoding is still limited due to the scarcity of the fluorescent dyes and their easy quenching. In addition, different color spectra emitted by multiple fluorescent dyes under the same excitation light may overlap each other, which limits the application of multiplexed encoding.

#### 3.1.2 Quantum dot encoding

**FIGURE 3:**
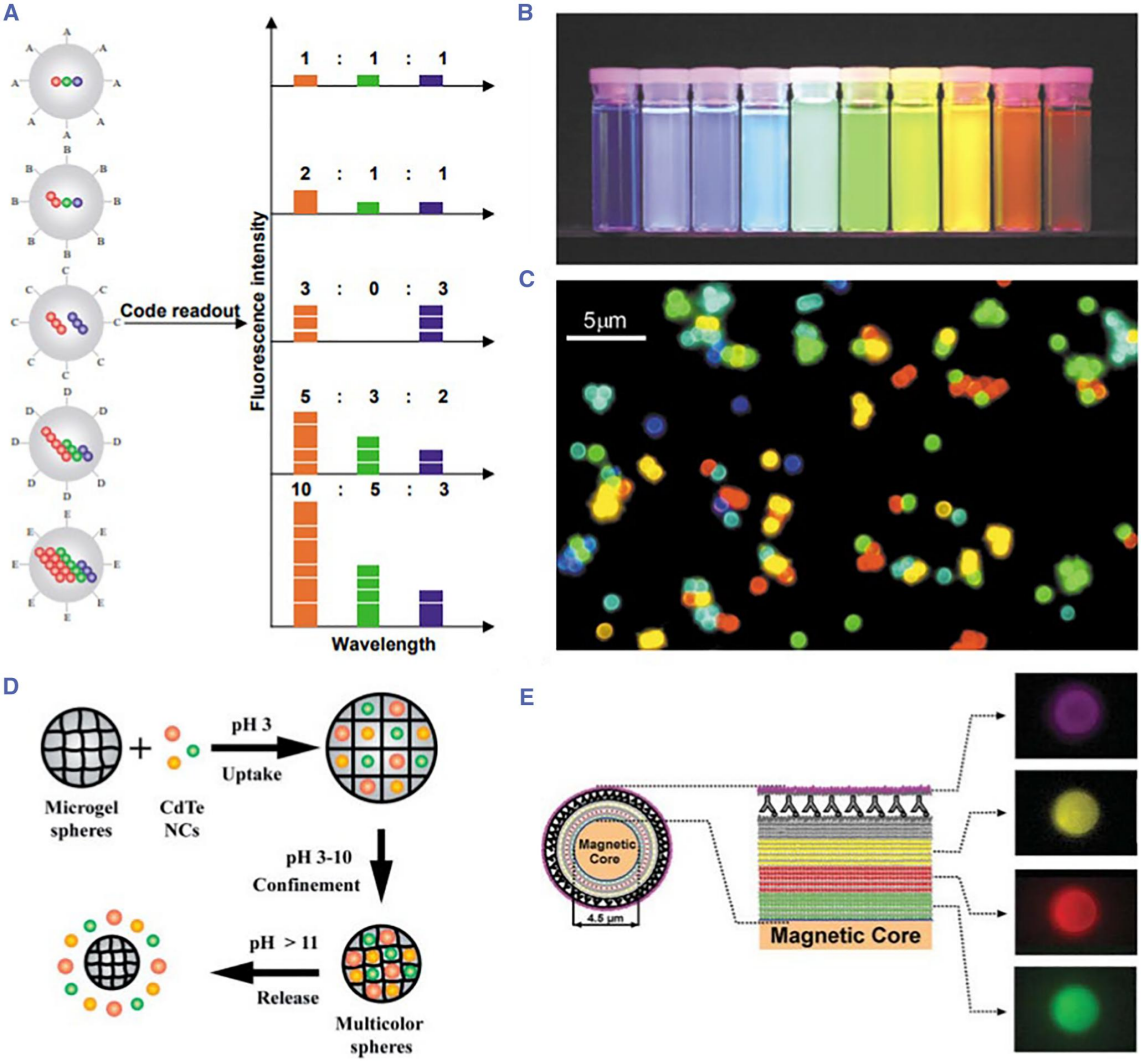
(A) Schematic diagram of QD microsphere encoding. Reproduced with permission.^40^ Copyright 2001, Springer Nature. (B) QD solutions with different emission light under the same excitation light. Reproduced with permission.^40^ Copyright 2001, Springer Nature. (C) Fluorescent image of polymer microspheres encoded by QDs. Reproduced with permission.^40^ Copyright 2001, Springer Nature. (D) Schematic diagram of QD‐encoded hydrogel microspheres. Reproduced with permission.^43^ Copyright 2005, John Wiley and Sons. (E) Schematic diagram of magnetic microspheres encoded with multicolor QDs. Reproduced with permission.^44^ Copyright 2007, Royal Society of Chemistry.

Quantum dots (QDs) are fluorescent semiconductor nanocrystals, normally composed of the nano‐particles of Group ii/vi or Group iii/v elements (Figure 3A–C),^40^ which have attracted increasing attention in recent years due to the avoidance of quenching and spectral overlap. QDs have the continuous excitation spectra, which can be obtained by adjusting the particle size. In addition, QDs have narrow emission spectra and broad excitation spectra. Based on the minimum spectral band gap, QDs with different particle sizes are simultaneously excited to obtain different colors of emission light. Compared with fluorescent dyes, QDs have better optical stability, as well as more ideal lifetime and fluorescence intensity, and have been widely used in the research of liquid‐phase encoded microcarriers.^41^

Nie et al. first proposed the concept of QD‐encoded microcarriers.^42^ By embedding QDs in polymer microspheres and tuning the type and strength of QDs, high‐throughput encoding microspheres were achieved. Wang et al. combined various water‐soluble QDs with hydrogel microspheres and prepared multi‐color encoded microspheres (Figure 3D).^43^ Robert Wilson et al. wrapped QDs on the surface of magnetic microspheres as encoding carriers for multiplexed analysis (Figure 3E).^44^ CdS, CdSe, CdTe, and ZnS are common materials for QD research, and they have strong biological toxicity. In the actual preparation and analysis of QDs, the overlapping fluorescence intensity impairs the QDs’ fluorescence encoding ability, and the corresponding solutions are limited to the restricted coding region or the improved decoding scheme. In addition, QD‐based biological detection requires the use of probe molecules, and the fluorescence of QDs may cause certain interference to the detection signal.

#### 3.1.3 Photonic crystal encoding

Photonic crystal (PhC) was proposed by Yablonovitch and John^45^, ^46^ in the discussion of the effect of periodic dielectric structure on the light propagation behavior in materials, that is, periodically changing the dielectric constant properties in the material space. The natural PhC structures can be widely found in nature,^47^ such as the butterfly wings and peacock feathers. Periodic permittivity permutations give them bright structural colors, hence the name structural color materials. Further, such structural color materials derived from a special physical structure present a stable optical property without signal quenching. Therefore, compared with the traditional encoding strategies, PhC encoding demonstrates a stable encoding and simple decoding process.^48^, ^49^, ^50^ More importantly, it has a large encoding capacity for high‐throughput detection.^51^

**FIGURE 4:**
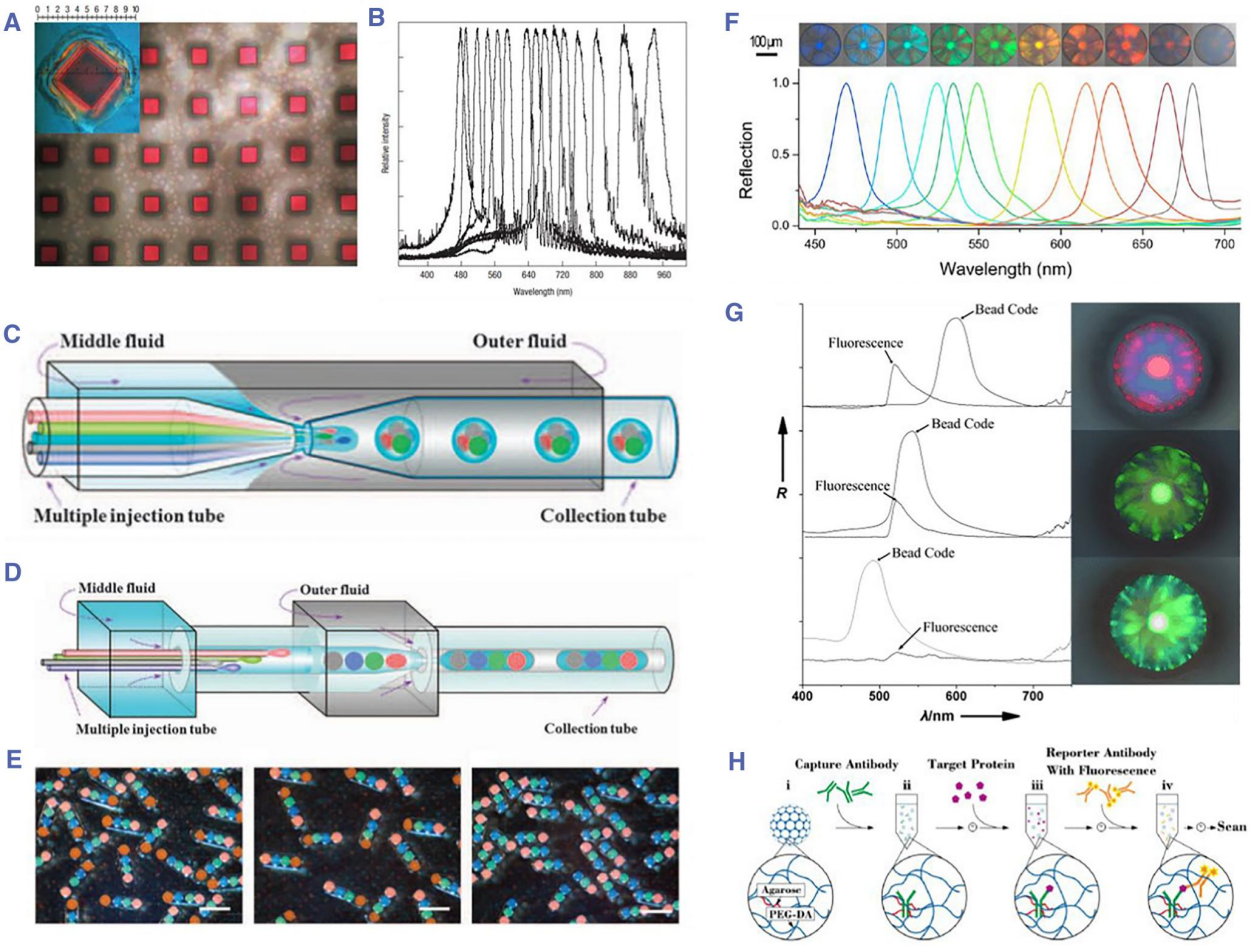
(A, B) Optical image and reflection peak of the monolayer porous silicon PhC carrier. Reproduced with permission.^52^ Copyright 2002, Springer Nature. (C) Schematic diagram of capillary microfluidic device for multi‐core double emulsion. Reproduced with permission.^54^ Copyright 2012, The Authors, published by Springer Nature. (D) Schematic diagram of capillary microfluidic device for rod‐shaped double emulsion. Reproduced with permission.^54^ Copyright 2012, The Authors, published by Springer Nature. (E) Rod‐shaped PhC barcodes. Reproduced with permission.^54^ Copyright 2012, The Authors, published by Springer Nature. (F) Optical image and reflection spectrum of PhC microsphere. Reproduced with permission.^55^ Copyright 2013, American Chemical Society. (G) Multiplexed biological detection based on colloidal crystal microspheres. Reproduced with permission.^56^ Copyright 2006, John Wiley and Sons. (H) Schematic diagram of bioanalysis based on hydrogel barcodes. Reproduced with permission.^57^ Copyright 2017, Elsevier.

PhC encoding was first proposed by Sailor et al. in 2001.^52^, ^53^ Flow‐encoding carriers based on PhC porous silicon flakes were set as the encoding elements for biological analysis (Figure 4A,B). Zhao et al. proposed PhC microspheres prepared using the microfluidic droplet method as the flow‐encoded microcarriers for biological multiplexed analysis (Figure 4C–H).^54^, ^55^, ^56^, ^57^ Due to the spherical symmetry of the PhC microcarrier, there is no difference in the detection of optical properties from any angle, thus ensuring the encoding stability of the microcarrier. Generally, the characteristic reflection peak of the PhC microcarrier follows Bragg’s Law. Under the normal incident conditions, the characteristic reflection peak is expressed as *λ* = 1.633*nd*_*n*_ (1), where *λ* is the diffraction wavelength, *n* is the average refractive index of the material, and *d*_*n*_ is the distance to the diffracting plane. According to this formula, PhC microspheres with different characteristic reflection peaks can be prepared by adjusting the size of colloidal nanoparticles.^54^

**FIGURE 5:**
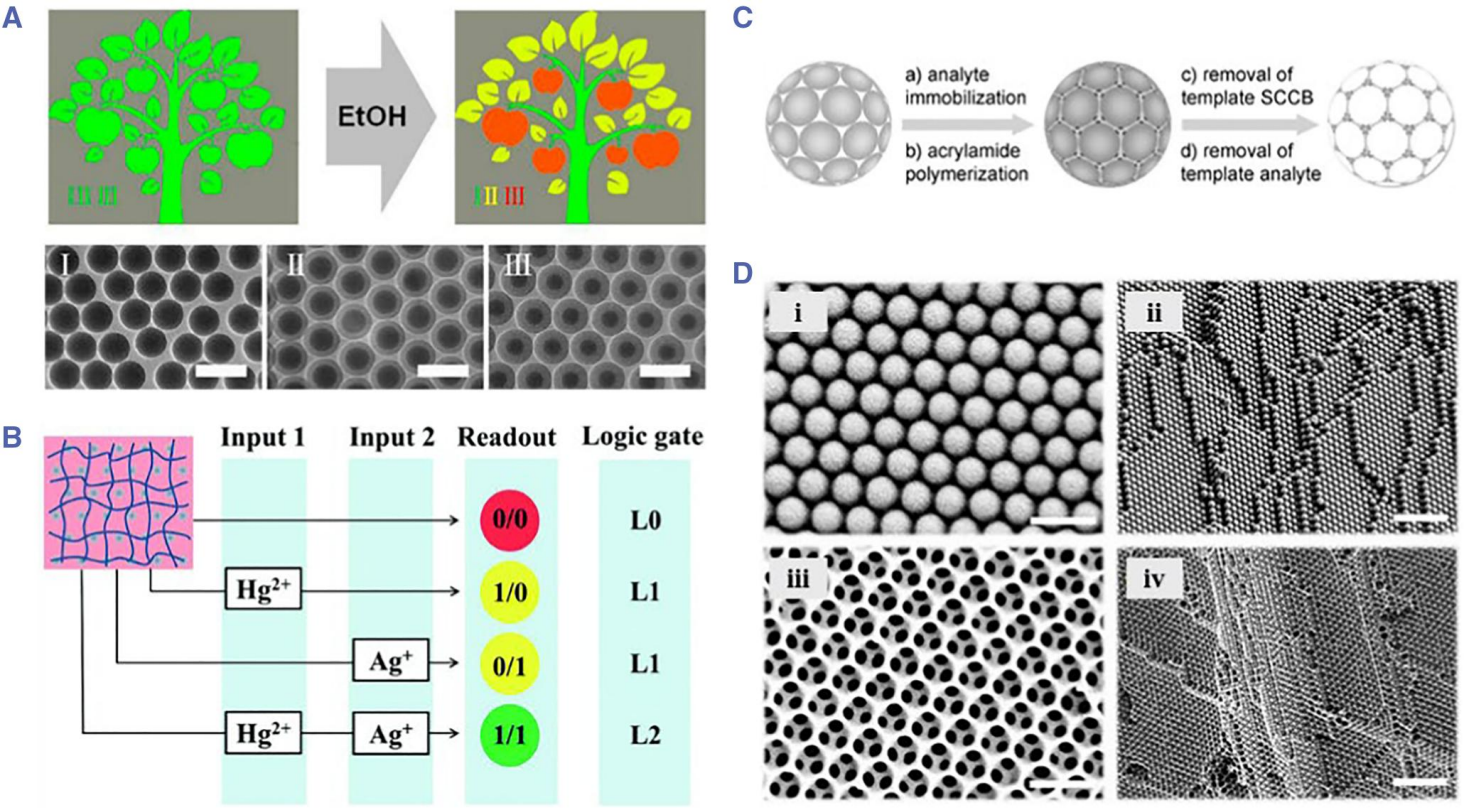
(A) Printed PhC sensor. Reproduced with permission.^58^ Copyright 2014, American Chemical Society. (B) PhC visualization sensor for biological detection. Reproduced with permission.^59^ Copyright 2015, Royal Society of Chemistry. (C) Schematic diagram of preparation of molecularly imprinted microcarriers with PhC structure. Reproduced with permission.^60^ Copyright 2009, John Wiley and Sons. (D) SEM characterization of PhC microcarrier. Reproduced with permission.^61^ Copyright 2017, American Chemical Society.

Zhao et al. used the self‐assembly PhC microspheres formed as the encoding microcarriers. With different sizes of nanoparticles, PhC microspheres were endowed with diverse structural colors, which could be used as “inks” for pattern printing (Figure 5A).^58^ When the refractive index of the PhC material changed, the structural color changed accordingly, which served as the basis for the development of vision sensors (Figure 5B).^59^ In addition, Zhao’s group has also carried out a series of related explorations based on the physical structure and optical properties of PhC microcarriers. PhC hydrogel microcarriers with an inverse opal structure were obtained by infiltrating the hydrogel into the gaps between the nanoparticles of the PhC microcarriers and etching the periodically ordered templates (Figure 5C,D).^60^, ^61^ According to the different material properties, various hydrogel microcarriers with environmental responsiveness have been prepared. Apart from the encoding effect of PhC, these microcarriers possess the specific response performance, such as *pH*, temperature, and ion strength.^62^ Besides, with the simple decoding process, such microcarriers are easy to be integrated with microfluidic analysis chips to achieve the high‐throughput biological detection.^63^, ^64^, ^65^, ^66^

#### 3.1.4 Raman spectrum encoding

**FIGURE 6:**
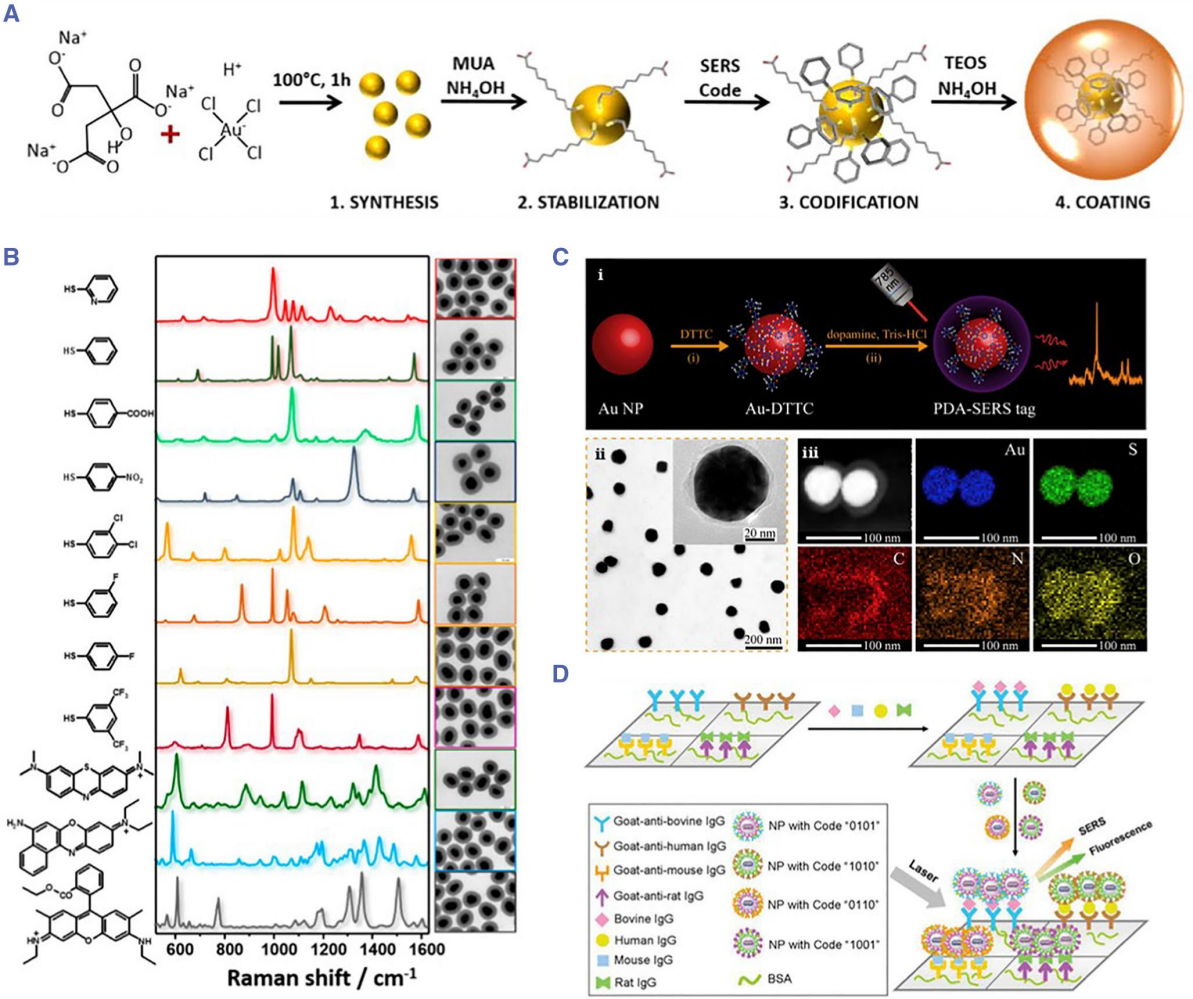
(A) Schematic diagram of the synthesis of SERS‐encoded nanoparticles. Reproduced with permission.^70^ Copyright 2015, American Chemical Society. (B) Spectrum of SERS‐encoded nanoparticles. Reproduced with permission.^70^ Copyright 2015, American Chemical Society. (C) Schematic diagram and characterization of SERS nanoparticles. Reproduced with permission.^71^ Copyright 2016, Elsevier. (D) Schematic diagram of multiplexed immunoassay based on SERS‐encoded microcarriers. Reproduced with permission.^72^ Copyright 2012, American Chemical Society.

Raman spectroscopy results from the inelastic scattering of molecular motion. In order to enhance the Raman spectral coding effect, surface‐enhanced Raman spectrum (SERS) is obtained by modifying precious metal on the surface of the object to be measured, and the signal intensity is significantly improved. In addition, the fabrication of nanogaps by photolithography can also enhance the signal of the Raman spectrum. The SERS‐encoded signals can be interpreted using Raman microscopy and Raman spectroscopy (Figure 6A–D).^67^, ^68^, ^69^, ^70^, ^71^, ^72^

Deoring et al. fabricated the surface‐enhanced Raman encoded nanorods using the interval‐deposition of gold and silver^73^ and obtained up to 1056 codes. Furthermore, when the molecular beacon probe was immobilized on the nanorods, the dye on one end of the probe was quenched by the noble metal nanorods. Therefore, the fluorescence signal decreased while the Raman signal increased. This encoding microcarrier was used for the multiple single nucleotide polymorphism (SNP) genotyping and viral RNA detection with unlabeled detection by Raman microscopy. However, in this work, the fluorescence signal still caused some interference to the SERS signal. To overcome this problem, Braeuer et al. developed a displacement differential Raman spectroscopy technique to effectively remove the fluorescence interference on Raman signals.^74^ In addition, Gambhir et al. reported the application of the simultaneous imaging in vivo using ten different SERS‐encoded microcarriers.^75^

Compared with the traditional fluorescence spectrum encoding method, the liquid‐phase biochip strategies based on Raman spectrum encoding has been well applied in recent years for the advantages of high spectral resolution, stable spectral peaks, and good signal reproducibility.^76^, ^77^

### 3.2 Non‐optical encoding

#### 3.2.1 Graphic encoding

**FIGURE 7:**
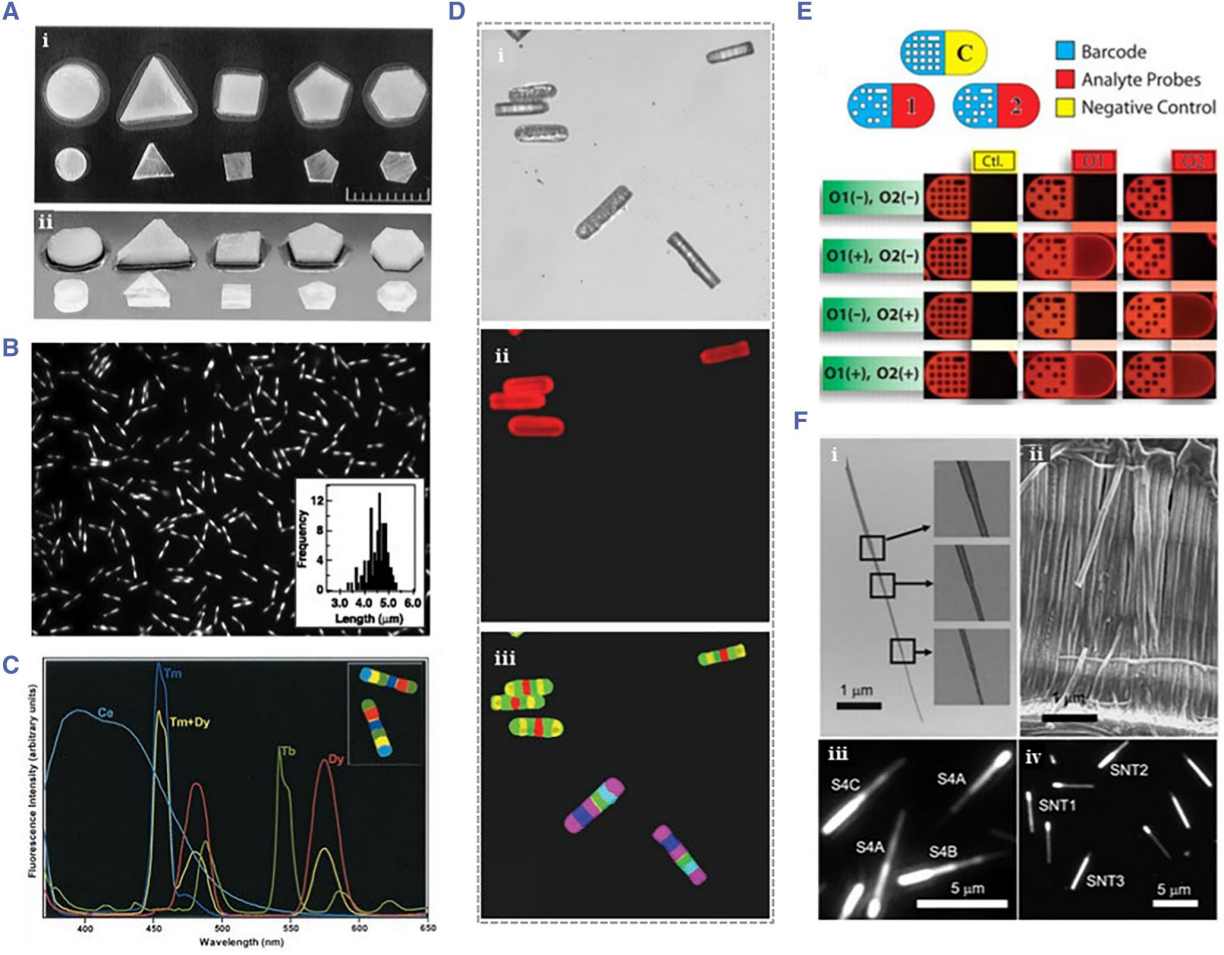
(A) Image of Euclidean shapes in dry and expansive states. Reproduced with permission.^78^ Copyright 2000, The National Academy of Sciences. (B) Optical image of barcode particle. Reproduced with permission.^81^ Copyright 2001, The American Association for the Advancement of Science. (C) Spectrally encoding color barcodes. Reproduced with permission.^82^ Copyright 2003, The National Academy of Sciences. (D) Image of barcode particles used in DNA hybridization. Reproduced with permission.^82^ Copyright 2003, The National Academy of Sciences. (E) Schematic diagram of two‐dimensional barcodes in multiplexed DNA detection. Reproduced with permission.^86^ Copyright 2007, The American Association for the Advancement of Science. (F) Electron microscope characterization and dark field optical characterization of carbon nanotubes. Reproduced with permission.^87^ Copyright 2007, American Chemical Society.

Graphic encoding includes shape encoding and barcode. The strategy of shape encoding is dominated by the preparation of microcarriers into the Euclidean‐ shaped polygons (Figure 7A).^78^ The preparation and decoding of microcarriers in this strategy are simple, but the encoding amount is limited. The barcode‐based graphic encoding strategy includes one‐dimensional barcodes and two‐dimensional barcodes, which were widely applied. There are many preparation methods for barcodes, such as electrochemical, electroplating, laser etching, and printing plate technology.^79^, ^80^ Natan et al. were the first to use the electrochemical way to prepare one‐dimensional metal barcodes with alternating distribution of gold and silver (Figure 7B).^81^ Under the incident light at 405 nm, the microcarriers exhibited bright silver and dark gold bands. Therefore, metal barcodes are prepared by adjusting the sequence and time of metal deposition. Lahiri et al. integrated the different rare earth elements into the glass rods in a preset order and generated colored barcodes under the excitation light irradiation (Figure 7C,D).^82^ Kim et al. prepared silicon nanoparticles using the etching method and immobilized DNA molecules on the substrates.^83^ The silicon particles were set as the graphic encoding microcarriers, and their microcarrier‐encoded information corresponding to the probe molecules was specifically identified and analyzed. Willson et al. prepared the hydrogel microcarriers and cap masks of different shapes using hydrogel polymerization for DNA multiplex analysis.^84^

Xiao et al. constructed two‐dimensional barcodes on ceramic substrates using laser etching.^85^ However, due to the large size of the microcarrier and the complicated preparation process, it has not been widely used. Doyle et al. mass‐produced two‐dimensional barcodes containing coding regions and probe regions using flow lithography, wherein the coding regions were encoded by microcarriers through patterns, and the probe regions were bioanalyzed by molecular reactions (Figure 7E).^86^ Since the encoding and the probe coupling belong to different regions, the encoding and detection mechanisms do not interfere with each other in the multiplex analysis, thereby improving the accuracy of decoding. Lee et al. synthesized various silicon nanotubes with different reflectance values by electroplating. Based on this encoding principle, the scheme was decoded using an optical microscope for high‐sensitivity protein molecular detection (Figure 7F).^87^

#### 3.2.2 Chemical encoding

**FIGURE 8:**
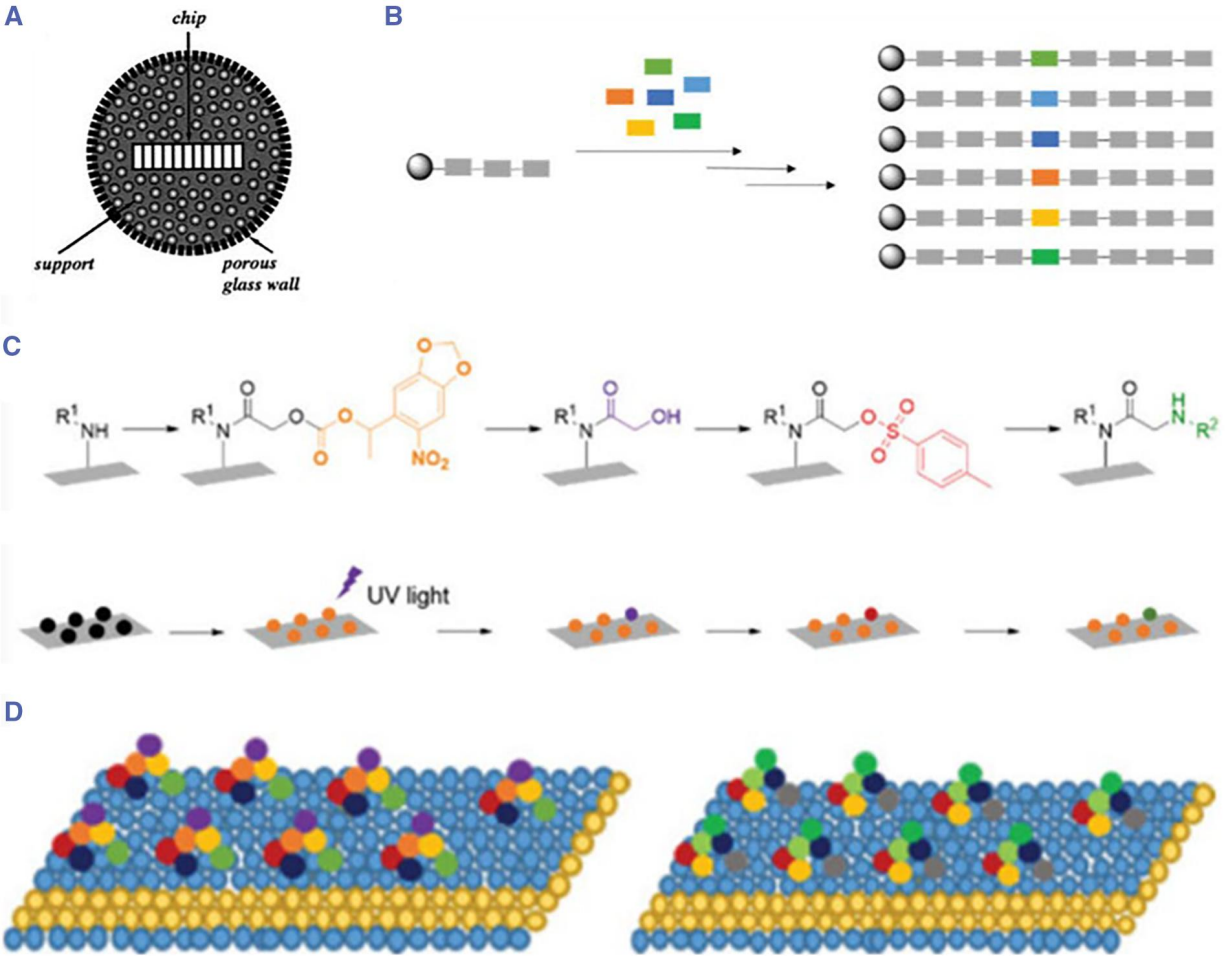
(A) Schematic diagram of polypeptide combination encoding microcarrier. Reproduced with permission.^90^ Copyright 1995, John Wiley and Sons. (B) OBOC chemical encoding. Reproduced with permission.^92^ Copyright 2021, Royal Society of Chemistry. (C) Schematic diagram for the assembly of peptide microarrays. Reproduced with permission.^92^ Copyright 2021, Royal Society of Chemistry. (D) Composite ring structure of nanosheets. Reproduced with permission.^93^ Copyright 2020, American Chemical Society.

Chemical encoding mainly adopts a combinatorial chemical encoding strategy. Brenner and Lener first proposed a method of alternating parallel synthesis of combinatorial libraries in 1992, called encoding combinatorial chemistry.^88^ This scheme can be used for oligonucleotide sequence encoding and synthesis. When each constituent unit is assembled on the solid‐phase carrier, the corresponding tags are also attached simultaneously or sequentially, and thus the assembled structural unit is recorded. With the increase of the compound library, the length of oligonucleotide label molecule also increases in parallel. Finally, the corresponding molecular structure information is identified according to the recorded labels. Lam first described the one bead one compound (OBOC) libraries.^89^ He found that multiple copies of distinct peptides presented on the bead after incubating with an amino acid. Numerous beads carrying the same compound were synthesized using the split‐pool method. At present, oligonucleotide labeling, polypeptide labeling, halogenated hydrocarbon labeling, and their derived mass spectrometry and X‐ray labeling methods have been used in chemical encoding (Figure 8A–D).^90^, ^91^, ^92^, ^93^ Though these methods have their own characteristics, they are often restricted and affected by some factors, such as the reaction conditions in the application. Moreover, these strategies are highly professional and technical, requiring sophisticated instruments and skilled operators.

#### 3.2.3 Electronic encoding

**FIGURE 9:**
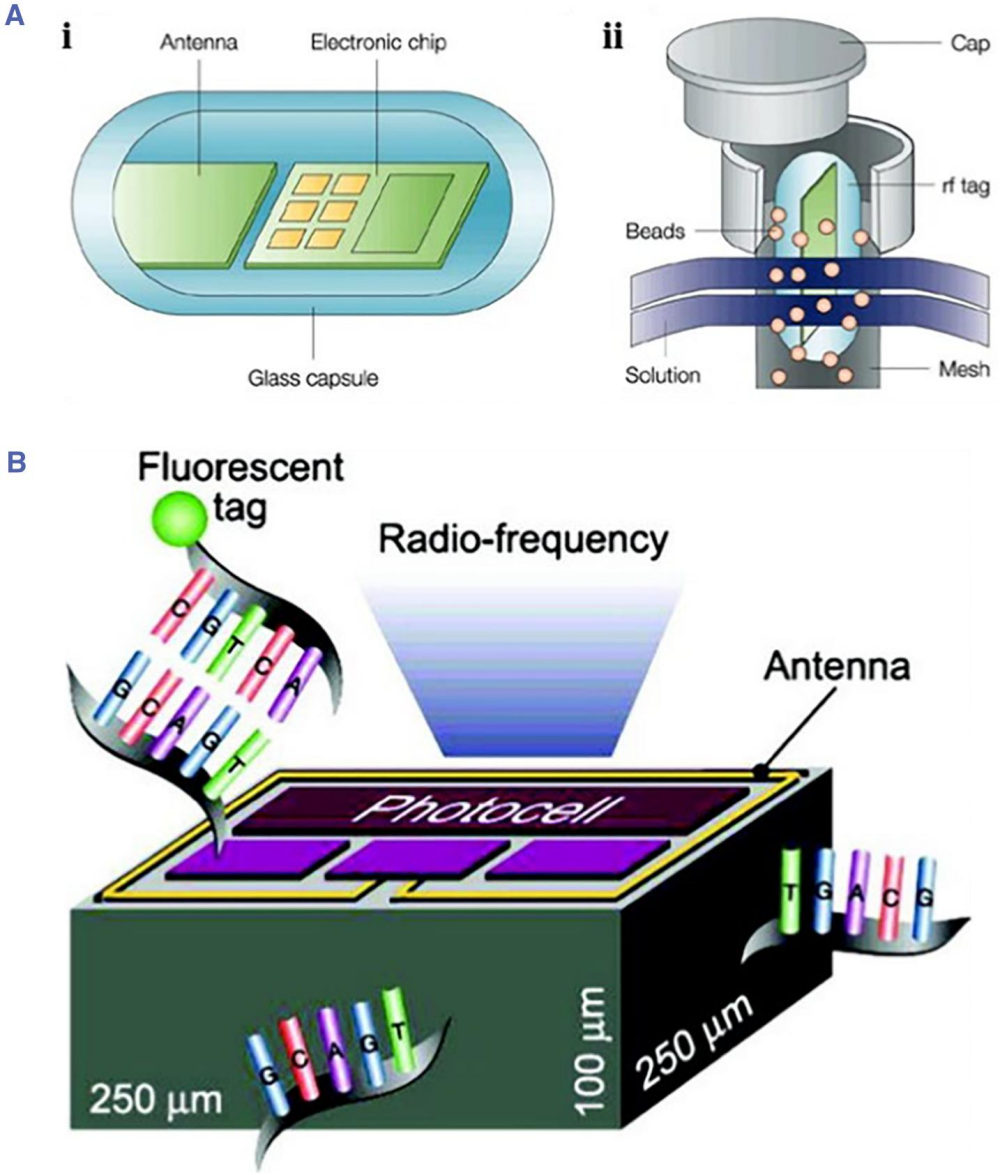
(A) Schematic diagram of electronic chip encapsulated with a glass capsule. Reproduced with permission.^94^ Copyright 2002, Springer Nature. (B) Schematic of electronic RF microchip. Reproduced with permission.^96^ Copyright 2006, John Wiley and Sons.

Electronic encoding utilized radio frequency (RF) memory tags to encode the combinatorial compound libraries.^94^, ^95^ As shown in Figure 9A, the RF memory tag consisted of an antenna and a microelectronic chip containing a laser‐etched identification code, and was encapsulated in a glass cavity. When the radio memory marker was close to the radio receiving device connected to the computer, the antenna received the electromagnetic wave signal sent by the radio receiving device and converted it into electrical energy to activate the microelectronic chip. After that, the microelectronic chip sent the identification codes to the radio receiving device in the form of an electromagnetic pulse. The receiving device read the codes and identified the tags. This approach has a large encoding amount and can improve the diversity level through a variety of fluorescent tags, and thus it also has applications in SNP and DNA detection. However, such electronic components are difficult to be widely used due to the large size after being encapsulated in the glass capsules.

Zbigniew Darzynkiewicz et al. constructed an electronic RF microchip, also known as a microtransponder (MTP). Each MTP provides a unique RF identification signal to match the chip to specific biomaterials (Figure 9B).^96^ Powered by photocells, with antennas transmitting signals, MTPs have been developed as analytical platforms in the fields of genomics and proteomics.^97^

#### 3.2.4 Phase change encoding

**FIGURE 10:**
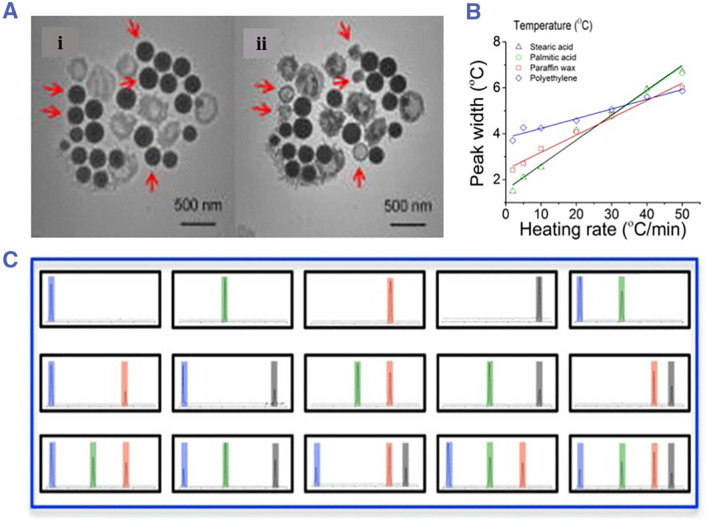
(A) Phase transition characterization of silicon‐coated lead‐bismuth nanoparticles. Reproduced with permission.^98^ Copyright 2009, AIP Publishing. (B) Phase transformation effect of different types of nanoparticles (indium, tin, bismuth, and indium Sn‐bismuth eutectic alloys). Reproduced with permission.^100^ Copyright 2014, Springer Nature. (C) Encoding and decoding of thermal responsive phase transition materials. Reproduced with permission.^100^ Copyright 2014, Springer Nature.

Phase change encoding is a new cryptic encoding method with a large amount of codes based on the phase‐change properties of nanomaterials. Due to temperature changes, these nanomaterials undergo physical properties, such as solid–liquid phase conversion. Therefore, phase change encoding is a typical thermal encoding strategy. Nanoscale‐encoded alloy microcarriers were prepared by mixing a variety of pure metals with different liquefaction temperatures (Figure 10A–C).^98^, ^99^, ^100^ During the heating process, the encoded alloy microcarrier gradually melted and was characterized by a differential scanning calorimeter (DSC). By using *n* kinds of pure metals, 2*n*‐1 kinds of microcarriers with different codes can be prepared. The metal is protected by a silica shell to prevent leakage after melting. In addition, some organic solids such as paraffin, polyethylene, stearic acid, and even palmitic acid can be used for phase change encoding. Polymer shells are used to encapsulate organic solids to avoid aggregation. These encoded microcarriers are decoded by heat and have high‐temperature resistance.^101^ Phase change materials are used in thermal encoding, no chemical reactions occur, and the encoding operation is simple and environmentally friendly.

### 3.3 Other encoding strategies

In addition to the above encoding strategy, there are also some properties such as size, density, and the encoding method of encoding elements.^102^, ^103^ Due to the small number of encodings and high preparation costs, the external application is limited. In addition, the combination of different encoding strategies can not only combine the advantages of various encoding strategies but also significantly increase the encoding amount. Therefore, in recent years, the hybrid encoding strategy has become one of the research hotspots in the multiplexed analysis. Genechip probe arrays use spatially patterned, light‐directed combinatorial chemical synthesis for specific high‐throughput genetic and cellular assays.^104^ Moreover, bead‐based high density arrays have been utilized for simultaneous characterization of multiple analytes.^105^ Luminex xMAP liquid chip technology is used for high‐throughput analysis of biomolecules by integrating fluorescence encoding, laser analysis, and signal processing.^106^, ^107^

## 4 BIOMEDICAL APPLICATIONS OF ENCODED MICROCARRIERS

### 4.1 Multiplexed biosensing

Multiplexed bioanalysis is an important research topic in modern biomedical research and clinical diagnosis. Microcarrier encoding and probe molecule immobilization are used to encode biomolecules so as to realize the high‐throughput multiplexed analysis, a reduced analysis time and sample consumption. Currently, biomolecular microarrays immobilized on solid substrates are the typical representatives of multiplexed bioanalysis platforms. With the introduction of the gene chip, a high‐throughput and high‐efficiency analysis strategy has been widely used in the bioanalysis field.^108^ Subsequently, the high‐throughput bioanalysis platforms such as protein chips and cell chips appeared.

Although solid‐phase microarray encoding is an important biomedical technology innovation of the 21st century, there are still inherent defects in the methodological principle. The position distribution of the microarray is fixed, and the diffusion speed of biomolecules to the lattice position affects the detection efficiency. In recent years, the application of mobile‐encoded microcarriers in biological multiplexing has received increasing attention.^109^, ^110^ Since the ideal fluidity, the mobile‐encoded microcarrier can fully react with the detected molecule in bioanalysis, which greatly improves the detection efficiency. As the floating vectors, mobile‐encoded microcarriers can identify the biomarkers quickly and reliably in the analytical systems. Therefore, mobile‐encoded microcarriers can be used as an ideal multiplexed biosensing scheme in bioanalysis.

**FIGURE 11:**
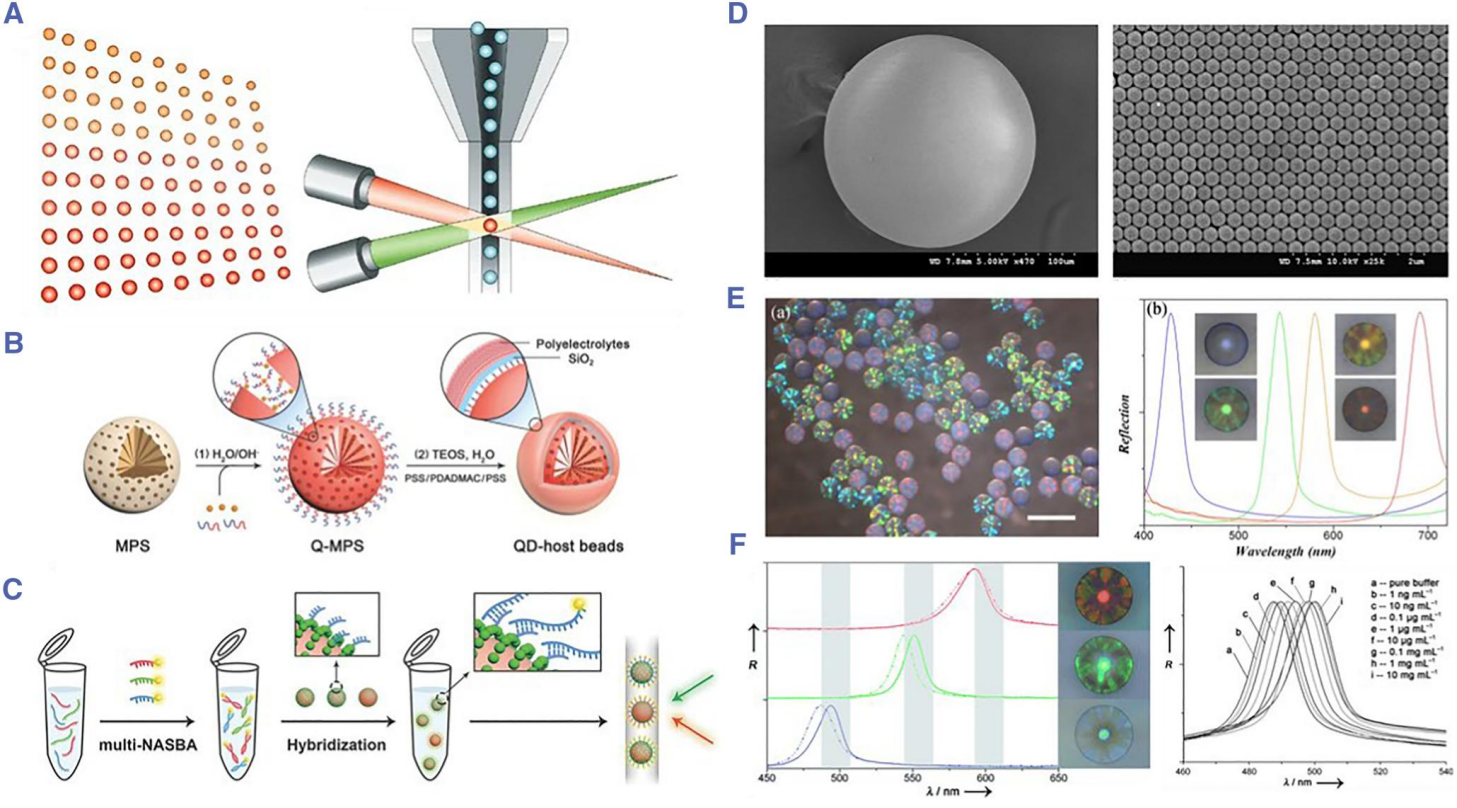
(A) Schematic diagram of fluorescent encoded microspheres and the Luminex XMAP detection system. Reproduced with permission.^94^ Copyright 2002, Springer Nature. (B) Schematic diagram of preparation of QD microbeads. Reproduced with permission.^115^ Copyright 2016, John Wiley and Sons. (C) Schematic diagram of nucleic acid multiplexed detection based on QD encoding. Reproduced with permission.^115^ Copyright 2016, John Wiley and Sons. (D) SEM images of colloidal crystal microspheres. Reproduced with permission.^119^ Copyright 2009, Elsevier. (E) Optical microscopic and spectral characterization of colloidal crystal microspheres. Reproduced with permission.^119^ Copyright 2009, Elsevier. (F) Optical characterization and biosensing of molecularly imprinted microcarriers. Reproduced with permission.^60^ Copyright 2009, John Wiley and Sons.

Luminex developed a high‐throughput analysis platform based on fluorescent encoded microspheres, which has successfully commercialized and launched XMAP technology products (Figure 11A).^111^, ^112^ The strategy used a mixture of fluorescent dyes to encode polystyrene microspheres and the amount of which was determined by the proportion of each dye used. In the detection, a solution containing fluorescent microspheres is excited by two different colored laser light, one representing the coded color of the microspheres and the other representing biomolecular information on the surface of the microspheres. A large number of encoding microcarriers with different characteristic fluorescence spectra can be obtained by adjusting the different ratios of fluorescent dyes and then covalently coupled to the capture probes for the specific biomarkers. For bioassays, trace samples are added to cocultures of encoded microspheres of various analytes, while the target biomarkers bind specifically to the probe molecules on the surface of microcarriers. High‐throughput detection reactions can be performed simultaneously in each biological reaction well. XMAP has been widely used in genotyping, cytokine analysis, HLA typing, and infection detection for flexible application and rapid detection. However, due to the scarcity of fluorescent dyes and photobleaching, there are few types of microcarriers prepared using this strategy. In addition, the interference between the encoding signal and the detection signal affects the encoding stability of the analysis system.

Compared with the traditional fluorescent dyes, QD encoding is more stable. By controlling the number of QDs and the proportion of fluorescent particles to obtain different fluorescence intensities, the encoding amount of the analysis system can be effectively enlarged.^113^, ^114^ Gu et al. doped the QDs into porous polystyrene microspheres and then linked the fluorescent particles through chemical coupling to prepare the composite encoding microspheres.^115^ The encoded microspheres were used for influenza virus detection and showed extreme sensitivity. However, the same problem with QDs and fluorescent dye encoding are that the detection signal is still susceptible to interference. Doyle et al. prepared graphically encoded microcarriers with miRNA probes for multiplexed detection of miRNAs in cell lysates, which greatly reduced detection time and cost due to the omission of complex purification and amplification steps.^116^ In addition, the microcarrier encoding of graphics as the encoding elements are stable and scalable in capacity.

Due to the PBG effect generated by the periodic ordered physical structure, PhC has significant advantages such as large encoding capacity and high stability, which makes it widely used in the biomedical field. In biosensors or biochips, biomolecules such as antibodies and nucleic acids are usually immobilized on the microcarrier surface as the probes to capture the targets in the sample. Zhao et al. used a droplet microfluidic chip to prepare the PhC microspheres with good monodispersity and controllable particle size and used them as the biomolecular microcarriers and their characteristic reflection peaks as the encoding elements to realize the multiplexed analysis of labeled molecules (such as tumor protein molecules) (Figure 11B–E).^115^, ^116^, ^117^, ^118^, ^119^ In the detection, specific probe molecules immobilized on the surface of different microcarriers were mixed in one tube so that multiple biomarkers can be detected simultaneously. The PhC‐based encoding mechanism is stable and does not interfere with the detection signals.

To further improve the detection sensitivity, an inverse opal microcarrier was fabricated using a PhC template for biosensing. Qian et al. immobilized antibodies on the inner surface of the three‐dimensional porous inverse opal PhC.^120^ When the corresponding antigen is encountered, the specific binding with the antibody can lead to the change of the thickness of the protein molecule layer on the inner surface of the porous structure, which in turn causes the change of the d_n_ in the crystal pores. Ozin et al. prepared a SiO_2_/PFS composite hydrogel film with the ferrocene silane (PFS) as the functional monomer, which could produce an obvious color change under an electric field.^121^ Next, they etched SiO_2_ to obtain a PFS‐only colloidal crystal hydrogel film, which significantly improved the response speed and the magnitude color change.^122^ The interaction of biomolecules can be quantitatively detected by the shift of crystal reflection peaks. Zhao et al. achieved the multiplex detection of tumor protein biomarkers based on three‐dimensional inverse opal PhC microspheres.^123^ He prepared the composite microspheres by injecting hydrogels into PhC microspheres for multiplexed detection. Composite microspheres can also be prepared by injecting hydrogel into PhC microspheres for multiplexed detection.^124^ This composite microcarrier has stable encoding characteristics and avoids the nonspecific adsorption of biomolecules, thus achieving high sensitivity and specificity for the detection of tumor targets.^125^, ^126^

In recent years, Zhao et al. combined molecular imprinting technology with a PhC encoding strategy to immobilize biomolecules on silica nanoparticles to prepare the microspheres using periodic ordered nanostructures of PhC as the structural templates (Figure 11F).^60^ Then, the acrylamide gel was filled into the microsphere space for polymerization, and the silica nanoparticles and biomolecules were removed to prepare the molecularly imprinted polymer microcarrier. Through the combination of biomolecules and recognition sites on the gel microspheres, the volume of the gel microspheres changes and the reflection peak shifts, thus realizing the qualitative and quantitative analysis of biomolecules. In addition, Zhao et al. took advantage of the bright structural color of colloidal hydrogel materials with great application potential in display. On the basis of previous research, it was proposed to use the membrane assembled by colloidal crystal microspheres as the template for hydrogel replication to fabricate an unbiased colloidal hydrogel membrane, which showed good application prospects in information display and electronic paper.^127^

### 4.2 Drug screening and delivery

**FIGURE 12:**
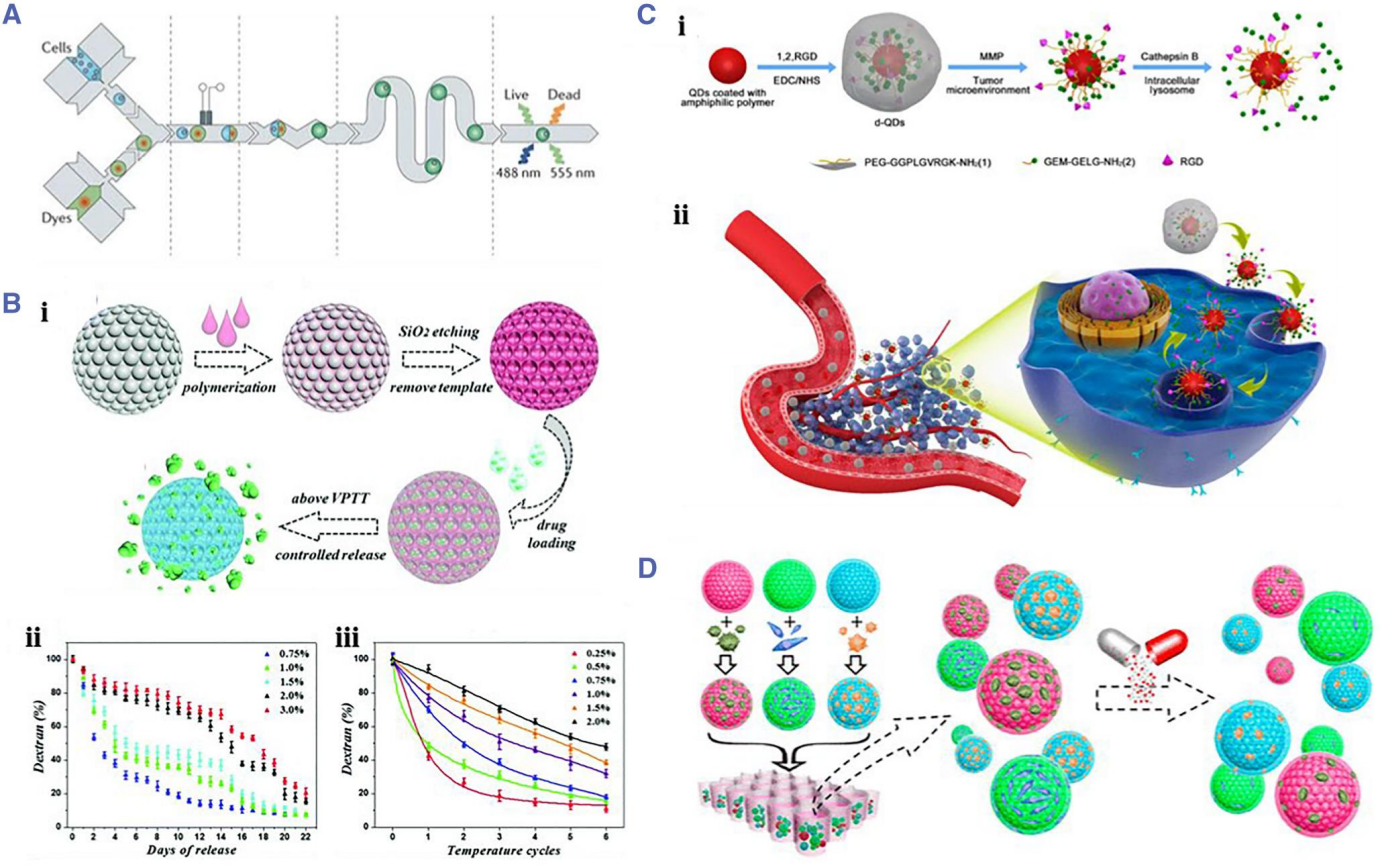
(A) Schematic diagram of droplet‐cell‐based drug screening. Reproduced with permission.^132^ Copyright 2012, Springer Nature. (B) Schematic diagram of drug carrier preparation and sustained‐release characterization. Reproduced with permission.^62^ Copyright 2015, Royal Society of Chemistry. (C) Schematic diagram of preparation and biological reaction of nanomedicine microcarriers. Reproduced with permission.^140^ Copyright 2017, American Chemical Society. (D) Schematic diagram of drug screening of colloidal crystal microspheres. Reproduced with permission.^141^ Copyright 2016, American Chemical Society.

With the development of modern medical technology, many pharmaceuticals and biomolecules have been developed for the prevention and treatment of diseases. Although with much progress, only a small number are used in clinical therapeutics due to their deficiency in clinical pharmacokinetic properties, such as the accumulation in nontarget tissues and organs, side effects, or high clearance rate.^128^, ^129^ Traditional administration methods are mainly oral and injection. Frequent administration is often required to maintain the drug concentration in the body, which is complicated and prone to overdose.^130^, ^131^ In order to solve these problems, the research on new drug delivery routes has attracted extensive attention. Particularly, a drug delivery system based on microcarriers is an important research progress (Figure 12A).^132^, ^133^, ^134^ The microcarrier drug delivery system can encapsulate the drug, avoid drug waste, and improve the utilization rate by controlling the speed and amount of drug release. At the same time, the small and constant drug dose reduces the toxic side effects of drugs and improves the use safety.^135^, ^136^ Further, encoded microcarriers can be used for drug screening and delivery due to their unique encoding elements and unique recognition properties.^137^, ^138^, ^139^

Zhao et al. proposed an n‐isopropylacrylamide (NIPAM) hydrogel inverse opal microcarrier as the drug delivery system (Figure 12B).^62^ NIPAM is a temperature‐responsive hydrogel that shrinks in volume above its phase transition temperature. Moreover, NIPAM is widely used in biochemical sensing, drug delivery, and tissue engineering due to its good biocompatibility. The inverse opal structure enables microcarriers with stable optically encoding properties for real‐time monitoring of pharmacokinetics. The interpenetrating pore structure provides channels for the load and release of drug molecules. When NIPAM inverse opal hydrogel is used for drug delivery, macromolecule substances were first loaded into the pores of the microcarriers through physical encapsulation. When the temperature is higher than the phase transition temperature of the hydrogel, the hydrogel shrinks and squeezes out the drug. Quantitative analysis of drug release can be achieved by detecting the displacement of the characteristic reflection peak of the microcarriers.

Ji et al. proposed a QD‐encoded targeted drug delivery microsphere (Figure 12C).^140^ The short peptide GELG that can be degraded by cathepsin B and the RGD peptide that promotes cellular internalization were modified on the surface of QDs, respectively. The microspheres were coated with PEG with one end linked to another short peptide GGPLGV RGK, which can be degraded by MMP‐9 to protect the microcarriers during the drug delivery and prolong the residence time in vivo. MMP‐9 is overexpressed in cancer tissue, and the short peptide linked to PEG has degraded after the microcarriers coupled this site. Thus, the PEG layer is destroyed, and the drugs and RGD peptides on the surface of the microspheres are exposed, promoting the cellular response to internalize the microspheres into the cells. Finally, cathepsin B degrades GEM microspheres for therapeutic effects.

In the drug toxicity experiments at the cellular level, Zhao et al. constructed core‐shell PhC microspheres, of which shells were methacrylic acid‐modified gelatin materials, which could simulate the three‐dimensional biochemical microenvironment in vivo to promote cell adhesion and growth.^141^ The cores of microspheres were the PhC structure, which provided a stable optical encoding platform to identify different cells during the monitoring of biochemical reactions (Figure 12D). Based on the encoding properties of PhC, microcarriers of hepatoma cells (HepG2) and monoclonal colon cancer cells (HCT‐116) were constructed for the cytotoxicity of the drug Tegafur. The results showed that the toxicity of Tegafur to HCT‐116 significantly enhanced under the action of HepG2 cells and fibroblasts (NIH‐3T3).

### 4.3 Cell culture and capture

At present, cell culturing is mostly based on cell adherent growth on the container substrate. Cell growth and metabolism were analyzed by observing the cell morphology and proliferation.^142^, ^143^ However, there are significant differences in cell growth between in vitro culture and in vivo settings, which may lead to significant differences in the cell morphology, differentiation, and interaction mechanism. Therefore, the usual in vitro two‐dimensional (2D) cell culture cannot fully simulate the in vivo environment.^144^ To solve this problem, the concept of three‐dimensional (3D) cell culture was proposed and received extensive attention from researchers.^145^ Compared with the traditional cell culture methods, the 3D cell culture strategy can provide cells with a spatial structure that is closer to the growth state in vivo, resulting in more accurate results.

**FIGURE 13:**
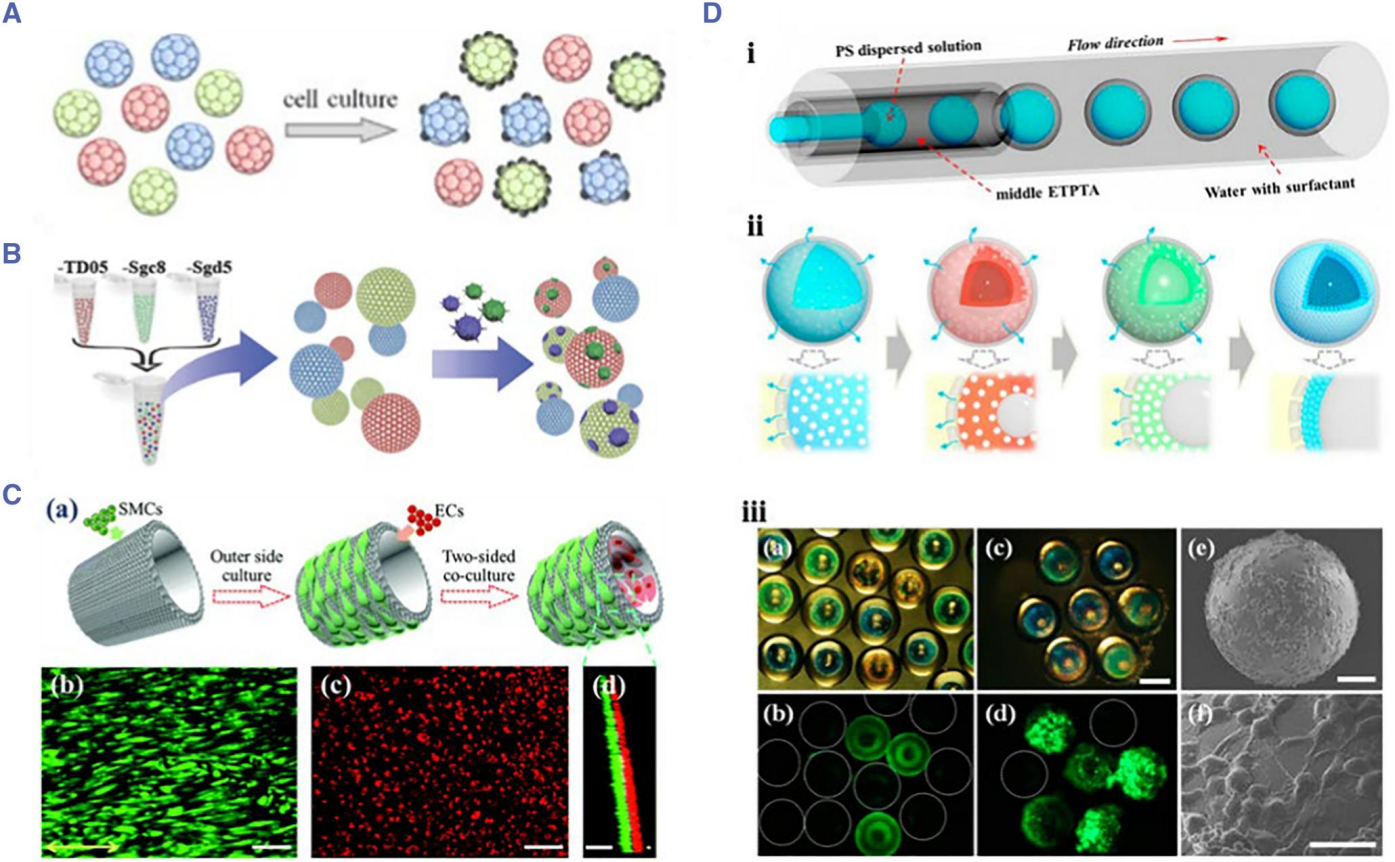
(A) Schematic diagram of encoded microspheres for evaluating the biological effects of hepatocellular carcinoma cells. Reproduced with permission.^148^ Copyright 2014, John Wiley and Sons. (B) PhC encoded microcarriers used to capture the circulating tumor cell CTC. Reproduced with permission.^149^ Copyright 2014, John Wiley and Sons. (C) Schematic diagram and characterization of tubular colloidal hydrogel for bionic blood vessel construction. Reproduced with permission.^153^ Copyright 2016, The Royal Society of Chemistry. (D) Schematic diagram of colloidal crystal self‐assembly and microcarrier characterization. Reproduced with permission.^154^ Copyright 2015, American Chemical Society.

Cellular microcarriers are suitable for adherent growth and have the ideal application value and potential in biomedical fields such as cell culture, functional measurement, and organ bionics.^146^ Currently, more and more materials and techniques are used to construct cellular microcarriers. Using microfluidic 3D printing technology, Soman et al. successfully encapsulated cells in GelMA hydrogel and prepared a series of size‐controllable hydrogel microspheres. Due to good biocompatibility, cells in these microspheres could remain viable for a long time.^147^ Zhao et al. carried out cell culture research based on PhC microcarriers (Figure 13A).^148^ PhC microspheres with uniform particle size were prepared using the droplet microfluidic technique, and multi‐cell culture was realized with the characteristic reflection peak as the stable optical encoding element. In this work, the silica used in the preparation of PhC microcarrier has no cytotoxicity, and the stability of spectral encoding will not be affected when the cells grow on the surface of the microcarriers. Microcarriers with different reflection peaks were constructed using different nanomaterials and then inoculated with the same cells for coculture. The biocompatibility of the materials is judged by the growth and proliferation of cells on the surface of microcarriers.

Cellular microcarriers with encoding features can be combined with biological probe molecules to capture the circulating tumor cells (CTCs) (Figure 13B).^149^ As a flow‐encoded microcarrier, PhC microspheres can efficiently capture CTCs. On the basis of this research, Zhao et al. fabricated the hydrogel‐infused PhC microcarrier and etched the periodic ordering templates of silica particles to obtain the encoding inverse opal microcarriers.^150^ The specificity of cellular targets can significantly improve the efficiency of biological responses when CTCs are captured by microcarriers. In addition, the group adopted the template replication method to obtain the tubular colloidal hydrogel and then stretched the pores of the tubular colloidal hydrogel to the spindle shape, thereby guiding the directional growth of cells.^151^, ^152^ The inner and outer walls of the tubular colloidal hydrogel were used to culture the endothelial cells and vascular smooth muscle cells, respectively. Meanwhile, the orientation was achieved.

The 3D culture of the above two types of cells has made a breakthrough and established the biological blood vessels, which is an important foundation for the development of organ chips (Figure 13C).^153^ In 3D cell culture, the common cell microcarriers can be precipitated by increasing cell density. To optimize this situation, the development of novel suspended cell microcarriers can effectively improve the fluidity and cell capturing efficiency of the encoding microcarriers. Zhao et al. fabricated the double‐shell encoded microbubbles using microfluidic technology and cavitation effect (Figure 13D).^154^ Microcapsule templates were prepared based on microfluidic technology. The shell of the template was a selectively permeable resin material, while the core was the aqueous solution of colloidal nanoparticles. Osmotic pressure induced bubbles in the microcapsules, thereby adjusting the overall density of the microcapsules to match the density of the liquid environment. Simultaneously, at the solid–liquid interface, the colloidal nanoparticles self‐assemble and finally form an ordered hexagonal tight‐packed colloidal crystal shell at the core of the microcapsule to prepare the double‐shelled microbubbles. By adjusting the number and color of the cores, the encoding capacity of the microcarriers can be expanded. This suspension microcarrier with the encoding properties can play a vital role in the related research of 3D cell culture.^155^, ^156^, ^157^

### 4.4 Bionic organ chips

**FIGURE 14:**
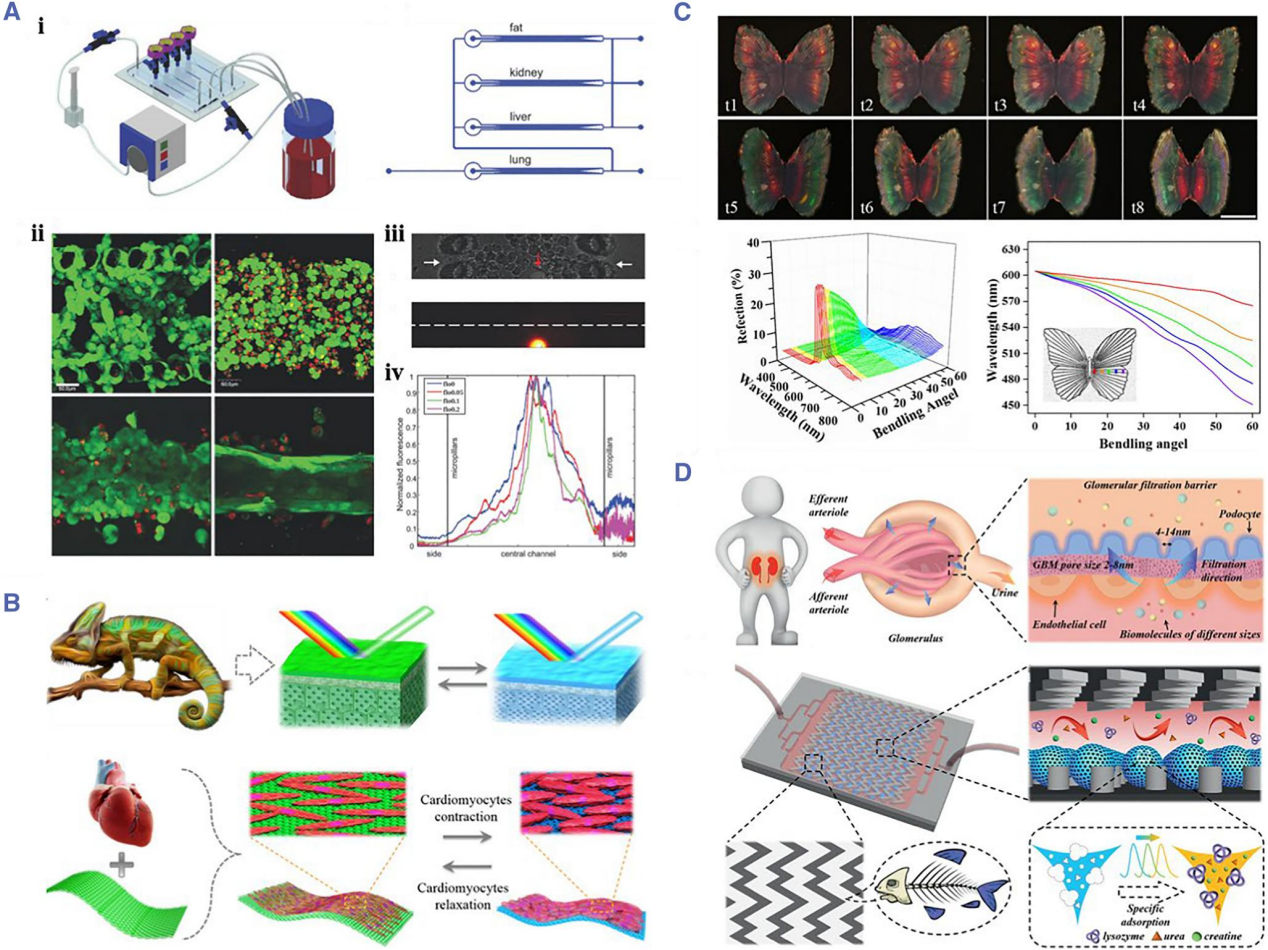
(A) A microchip that integrates a variety of cells to simulate the functions of human organs. Reproduced with permission.^158^ Copyright 2009, Royal Society of Chemistry. (B) Schematic diagram of preparation of bionic structural color hydrogel. Reproduced with permission.^159^ Copyright 2018, The Authors, published by the American Association for the Advancement of Science. (C) Optical characterization of myocardial actuated colloidal hydrogels. Reproduced with permission.^159^ Copyright 2018, The Authors, published by the American Association for the Advancement of Science. (D) Schematic diagram of biomimetic kidney cleaning with a molecularly imprinted microcarrier. Reproduced with permission.^160^ Copyright 2020, John Wiley and Sons.

In modern medical research, based on ethical and safety considerations, there is growing opposition to animal models and human trials. A bionic organ chip is a microsystem in which various cells are integrated in a microchip through microprocessing, thereby simulating the functions of human organs (Figure 14A).^158^ This strategy overcomes the defects of traditional cell culture in vitro and animal experiments, realizes the simulation of the in vivo environment, reduces the experimental costs and ethical disputes, and is expected to become a novel tool for drug development.

Zhao et al. took the lead in using templates to prepare the oriented PhC films and repeatedly prepared colloidal crystal structures for inducing the directional growth of cardiomyocytes (Figure 14B,C).^159^ When cultured on such hydrogels, cardiomyocytes were found to grow oriented. The contraction of cardiomyocytes can drive the motion of the hydrogel and realize the function of a soft robot. Furthermore, based on the encoding properties of the hydrogel, the shrinkage can lead to structural color changes. The growth and proliferation of cells can be roughly understood by analyzing their color and spectral changes. Therefore, the bionic chip strategy is expected to be applied to the construction of cardiac chips and the development of cardiac drugs.

PhC microcarrier combined with molecular imprinting technology provides a new idea for biosensing. Zhao et al. developed a novel fishbone microfluidic chip equipped with the hierarchical molecularly imprinted inverse opal microspheres, which was used for the bionic kidney to remove the excess biomolecules in blood with high efficiency and specificity (Figure 14D).^160^ The periodic macropores of the inverse opal microcarrier and the pores formed by molecular imprinting were used to form a hierarchical pore structure, which realizes the efficient and specific adsorption of biomolecules. Meanwhile, the characteristic structural color change of the microsphere could also be used as an indicator to reflect the adsorption process.^161^, ^162^, ^163^ The microspheres were integrated into the fishbone microfluidic chip, and the removal efficiency of blood molecules is further improved through the special flow channel design. The research has shown that the bionic kidney chip has high adsorption capacity and real‐time detection capability. The ideal reusability and biosafety present good prospects and application potentials in clinical blood purification and artificial kidney construction.

As mentioned above, we summarized the encoding strategies of microcarriers to clearly demonstrate their application and research progress in the biomedical field (Table 1).

**TABLE 1:** Encoding microcarrier strategies and the biomedical applications

Strategies	Mechanism	Biomedical applications	References
Optical encoding			
Fluorescent dye encoding	Fluorescent dyes are physically or chemically modified on the surface of microcarriers for the identification of the fluorescent types and the signal intensity detection.	High‐throughput biological assays	^34^, ^35^, ^36^, ^37^, ^38^, ^39^, ^94^, ^111^, ^112^
Quantum dot encoding	As the fluorescent semiconductor nanocrystals, quantum dots have the characteristics of broad and continuous excitation spectrum distribution.	Bioanalysis based on nano‐fluorescent labeling, drug delivery	^40^, ^41^, ^42^, ^43^, ^44^, ^113^, ^114^, ^115^, ^140^
Photonic crystal encoding	The periodic ordered nanostructures derived the structural colors, as the encoding elements of the photonic crystal, both stable encoding and simple decoding advantages.	High throughput bioanalysis based on the liquid phase encoding strategy, drug screening and delivery, cell 3D culture and CTC capture, bionic organ chip	^45^, ^46^, ^47^, ^48^, ^49^, ^50^, ^51^, ^52^, ^53^, ^54^, ^55^, ^56^, ^57^, ^58^, ^59^, ^60^, ^61^, ^62^, ^63^, ^64^, ^65^, ^66^, ^116^, ^117^, ^118^, ^119^, ^135^, ^136^, ^137^, ^138^, ^139^, ^140^, ^141^, ^159^, ^160^, ^161^, ^162^, ^163^
Raman spectrum encoding	Raman spectra generated by inelastic scattering of molecular motion, coupled with signal enhancement strategies, have the stable peak spectra and high resolution.	Biomedical imaging and biomolecular detection	^67^, ^68^, ^69^, ^70^, ^71^, ^72^, ^73^, ^74^, ^75^, ^76^, ^77^
Non‐optical encoding			
Graphic encoding	The microcarriers are prepared into different Euclidean graphics or barcodes and then decoded using the optical method.	Composite analysis of biomolecules	^78^, ^79^, ^80^, ^81^, ^82^, ^83^, ^84^, ^85^, ^86^, ^87^, ^115^, ^116^
Chemical encoding	Combinatorial libraries are ynthesized alternately in parallel, known as combinatorial chemistry encoding.	Biomolecules labeling and the derived mass spectrometry analysis	^88^, ^89^, ^90^, ^91^, ^92^, ^93^
Electronic encoding	The antenna coupled with the microelectronic chip encodes the combinatorial compound libraries	Genomics and proteomic analysis	^94^, ^95^, ^96^, ^97^
Phase change encoding	An encoding strategy based on phase transition characteristics of nanomaterials.	Biomolecules encoding and analysis	^98^, ^99^, ^100^, ^101^

## 5 CONCLUSION

Multiplexed analysis based on the encoded microcarriers is a new strategy of biological analysis and has been widely used in the biomedical field. The flow encoding mechanism of the microcarrier makes it more flexible in the floating encoding system, thus improving the reaction efficiency and detection sensitivity. In clinical diagnosis, the encoded microcarriers synchronously detect and analyze the multiple biomarkers by coupling probe molecules. After modification or construction, the encoding microcarrier can be used for cell culture and capture, drug screening, and delivery. Particularly, the visualized encoding effect of the microcarriers can also be involved in the biological effect characterization of bionic organ chips. Thus, as a flexible technology platform, the encoding strategies can be developed according to practical analysis and present a wide application in the biological analysis and biomedical field. However, due to the different encoding mechanisms, there are corresponding challenges from the encoding capacity to the bioanalytical performance. The photobleaching effect and signal interference can be caused by fluorescence encoding, while graphic encoding has a limited encoding capacity and a high preparation cost. All these problems affect the application of the encoding microcarriers. With the continuous development of the novel encoding microcarrier and further exploration of the practical applications, encoding microcarriers with better performance will be increasingly applied in the biomedical field.

## AUTHOR CONTRIBUTIONS

Yefei Zhu and Dagan Zhang conceived the topic of this review; Xiaowei Wei wrote the manuscript; Zhuxiao Gu and Yixuan Shang revised the content.

## CONFLICT OF INTEREST STATEMENT

The authors declare no conflict of interest.
